# Molecular and morphological characterisation of *Longidoruspolyae* sp. n. and *L.pisi* Edward, Misra & Singh, 1964 (Dorylaimida, Longidoridae) from Bulgaria

**DOI:** 10.3897/zookeys.830.32188

**Published:** 2019-03-14

**Authors:** Stela S. Lazarova, Milka Elshishka, Georgi Radoslavov, Lydmila Lozanova, Peter Hristov, Alexander Mladenov, Jingwu Zheng, Elena Fanelli, Francesca De Luca, Vlada K. Peneva

**Affiliations:** 1 Institute of Biodiversity and Ecosystem Research, Bulgarian Academy of Sciences, 2 Y. Gagarin Street, 1113 Sofia, Bulgaria Institute of Biodiversity and Ecosystem Research, Bulgarian Academy of Sciences Sofia Bulgaria; 2 Laboratory of Plant Nematology, Institute of Biotechnology, College of Agriculture and Biotechnology, Zhejiang University, Hangzhou 310058, China Zhejiang University Hangzhou China; 3 Istituto per la Protezione Sostenibile delle Piante, Consiglio Nazionale delle Ricerche (CNR), Bari, Italy Istituto per la Protezione Sostenibile delle Piante Bari Italy

**Keywords:** 18S rDNA, Bayesian inference, croplands, D2-D3 28S rDNA, ITS1-5.8S-ITS2, morphology, phylogeny

## Abstract

*Longidoruspolyae***sp. n**., a bisexual nematode species found in the rhizosphere of pear tree (*Pyruscommunis* L.), is described and characterised using an integrative approach. The new species has a female body length of 6.8–9.1 mm; a comparatively long odontostyle (114.0–127.5 μm); a narrow lip region (14.0–15.5 μm), anteriorly flattened and almost continuous with the body profile; pocket-like amphidial pouches long, deeply bilobed, and slightly asymmetrical, a guide ring at 37–42 μm from the anterior end; normal arrangement of pharyngeal glands; and a short bluntly rounded to hemispherical tail. Four juvenile stages identified: the first stage with a digitate tail, and the second and subsequent stages with a bluntly rounded tail. Males have one adcloacal pair and a row of 10 or 11 single ventromedian supplements; spicules 71.0–74.5 μm long. Based on morphometric data, the new species belongs to a group of species spread over Europe (*L.arthensis*, *L.silvae*, *L.uroshis*,), Iran (*L.kheirii*), and Syria (*L.pauli*), which share common characters such as amphidial fovea, lip region and tail shapes, similar odontostyle and body length, and similar first-stage juvenile tail shape. Codes for identifying the new species are A5, B2, C34, D3, E3, F45, G12, H1, I2, J1, K7. The phylogenetic analysis based on D2-D3 expansion domains of the rRNA gene revealed that the new species has the closest relationships with *L.athesinus* from Italy and three unidentified *Longidorus* spp. from USA (*Longidorus* sp. 1, *Longidorus* sp. 2, and *Longidorus* sp. 6). New morphometric and molecular data (18S rRNA gene, ITS1-5.8S-ITS2 regions and D2-D3 28S rRNA gene sequences) for three populations of *L.pisi* from Bulgaria were obtained and variations between populations are discussed.

## Introduction

*Longidorus* Micoletzky, 1922 is the second most diverse genus within family Longidoridae (Thorne, 1935) Meyl, 1961 occurring in all continents except Antarctica. At present, it contains 170 species (Barsalote et al. 2018; Gharibzadeh et al. 2018; Xu et al. 2018). So far, 16 species have been recorded in Bulgaria (Peneva et al. 2012, 2013). During the study of longidorids in croplands with organic and conventional management, an unknown species was recovered from the rhizosphere of a pear tree in north-central Bulgaria. Further, several populations of *L.pisi* Edward, Misra & Singh, 1964 were found in a tobacco field and two vineyards from the southwestern part of the country. *Longidoruspisi* was originally described from the rhizosphere of *Pisumsativum* L. growing at the Allahabad Agricultural Institute (now Sam Higginbottom University of Agriculture) in Uttar Pradesh, India (Edward et al. 1964). It was subsequently reported from various countries in Asia and Africa. *Longidoruslatocephalus* Lamberti, Choleva & Agostinelli, 1983, a very similar species to *L.pisi*, was described from southwestern part of Bulgaria where it was associated with various crops. Choleva et al. (1991) proposed that *L.latocephalus* is a junior synonym of *L.pisi*. However, Navas et al. (1993), using the same data and those of Brown et al. (1982), carried out comprehensive analyses and distinguished two separate groups of *L.latocephalus* (8 populations from Bulgaria) and *L.pisi* (3 populations from India, 2 from South Africa, and 1 from Malawi). As a result, the taxonomic status of these two species has been debated in several subsequent studies (Robbins et al. 1995; Lamberti et al. 1996, 1997; Chen et al. 1997). Although there are observed differences in certain metrical data and amphid shape, Loof and Chen (1999) accepted the synonymy of *L.latocephalus* and *L.pisi* in their revised polytomous key. In three recent studies, sequences of D2-D3 28S rRNA or the *cox*I gene for populations from Greece, South Africa, and Iran have been provided, but, only the last population was characterised with morphological and morphometric data (He et al. 2005; Pedram et al. 2012; Palomares-Rius et al. 2017).

The present study aims to characterise morphologically and molecularly: i) an unknown species of genus *Longidorus* and ii) populations of *L.pisi* from Bulgaria, and iii) to evaluate these species’ phylogenetic relationships by using 18S rRNA and D2-D3 expansion domains of the 28S rRNA genes.

## Materials and methods

### Sampling, nematode isolation and processing

The *Longidorus* specimens examined originated from various croplands in Bulgaria: Balgarene village (private garden, *Pyruscommunis* L. tree), Petrich (a small field of *Nicotianatabacum* L.), and two vineyards, near Sandanski (Polenitsa village, small-scale management) and Kromidovo village (organic farm). Nematodes were isolated from soil samples by a decanting and sieving technique (Cobb 1918). The recovered *Longidorus* specimens were heat killed at 55 °C for 2 minutes, fixed in a 4% formalin/1% glycerol mixture, processed to anhydrous glycerol (Seinhorst 1959), and mounted on glass microscope slides. Drawings were prepared using an Olympus BX51 compound microscope with differential interference contrast (DIC). Microphotographs and selected measurements were taken with an Axio Imager.M2 Carl Zeiss microscope, digital camera (ProgRes C7), and CapturePro 2.8 software (Jenoptic). Measurements were made using a system of a light microscope (Olympus BX41), digitising tablet (CalComp Drawing Board III) and Digitrak 1.0f programme (Philip Smith, the John Hutton Institute, Dundee, UK). The alpha-numeric codes of polytomous identification key of the genus *Longidorus* (Chen et al. 1997) and modified partial polytomous key (Peneva et al. 2013) were used for the morphological species delimitation.

### DNA extraction, amplifications and sequencing

The genomic DNA extraction, amplification, and sequencing of single female specimens from three populations of *L.pisi* were carried out independently in two laboratories: one at the Institute for Sustainable Plant Protection, Bari, Italy and two at the Institute of Biodiversity and Ecosystem Research, Sofia, Bulgaria (IBER-BAS). Protocols used in both laboratories are presented previously (Groza et al. 2017). DNA analyses of *L.polyae* sp. n. were done in the IBER-BAS. One female, one male, and two first-stage juveniles were used for DNA extraction, amplification, and sequencing. The amplified products were sequenced by Eurofins MWG Operon, Germany. Sequences were deposited in GenBank with the following accession numbers: MK172047 and MK172049 for 18S rRNA gene of *L.polyae* sp. n. and *L.pisi*, respectively, MK172046 for D2-D3 expansion domains of 28S rDNA of the new species, and MK172048 and LR032064-65 for *L.pisi*. The partial 18S-ITS1-5.8S-ITS2 ribosomal segment was also sequenced for one population of *L.pisi* (LR032063, Petrich).

### Sequence and phylogenetic analyses

The 18S and D2–D3 expansion segments of the 28S rRNA gene sequences were compared with those of other nematode species deposited in the GenBank database using the BLASTn similarity search tool. The homologous sequences nearest to those of the new species were aligned using the GUIDANCE2 Server (http://guidance.tau.ac.il/) with default parameters (Sela et al. 2015) and manually trimmed and edited in Mega 6 (Tamura et al. 2013). Pairwise sequence identities/similarities were computed using the Sequence Manipulation Suite online (http://www.bioinformatics.org/sms2/) (Stothard 2000). The Bayesian Inference (BI) algorithm implemented in MrBayes 3.2.5 was used for phylogenetic relationships reconstructions (Huelsenbeck and Ronquist 2001; Ronquist et al. 2012). For further details, see Lazarova et al. (2016).

## Results

### Longidorus polyae

Taxon classificationAnimaliaDorylaimidaLongidoridae

Lazarova, Elshishka, Radoslavov & Peneva sp. n.

http://zoobank.org/7BE5AED3-2352-4039-AFC5-4E0D2AB42C49

#### Description.

**Figure 1.**
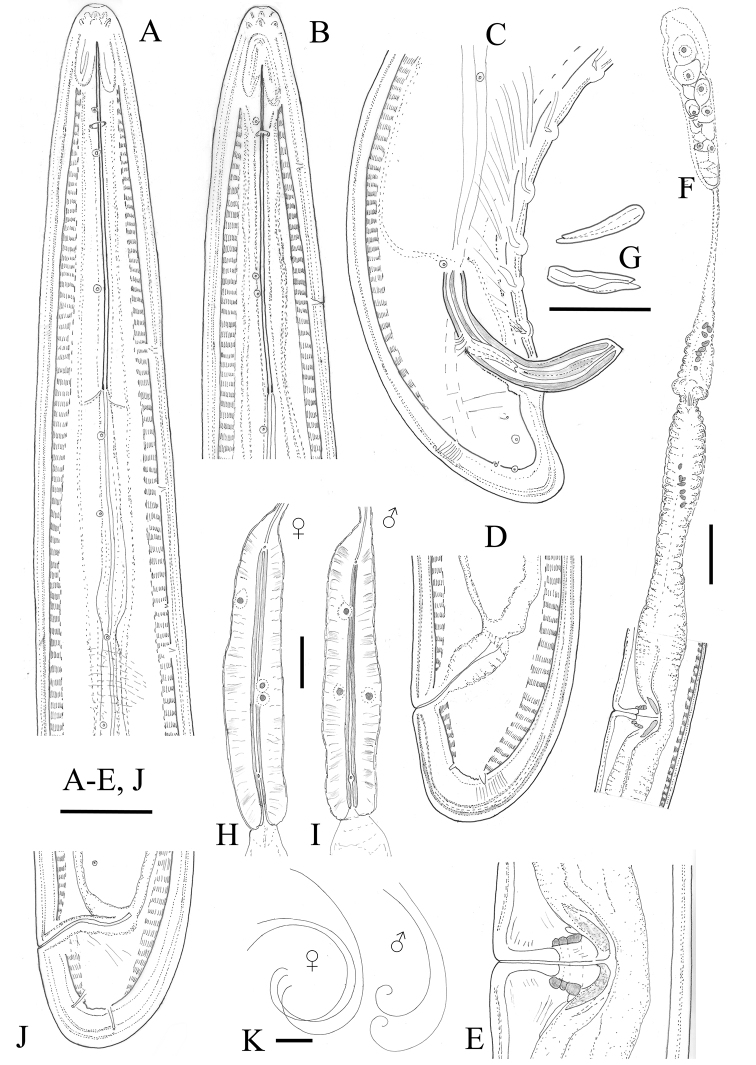
*Longidoruspolyae* sp. n. **A, B** anterior region **C** posterior region **D, J** variations in tail shape **E** vagina **F** anterior genital branch **G** lateral pieces **H, I** female and male pharyngeal bulb **K** variations in female and male habitus shapes. Female: all except **C, G** and **I**. Scale bars: 25 μm (**A–E, J, G**); 50 μm (**F**); 1 mm (**K**).

**Figure 2.**
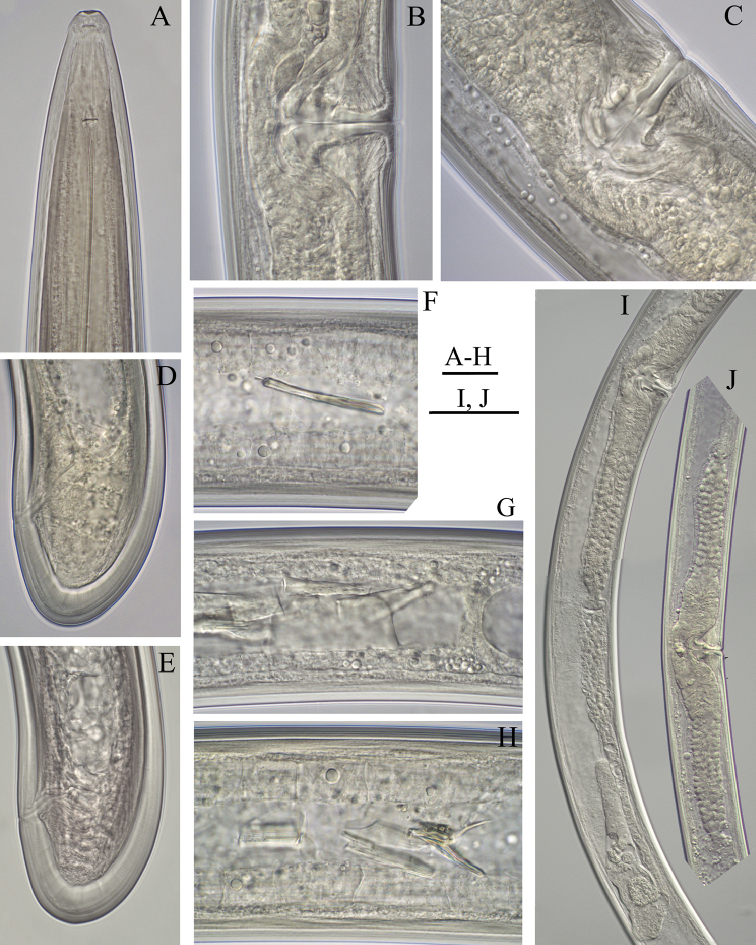
*Longidoruspolyae* sp. n., female **A** anterior region **B, C** variations in vagina **D, E** variations in tail shape **F–H** intestine inclusions **I** posterior genital branch **J** part of reproductive system. Scale bars: 20 μm (**A–H**); 100 μm (**I, J**).

**Figure 3.**
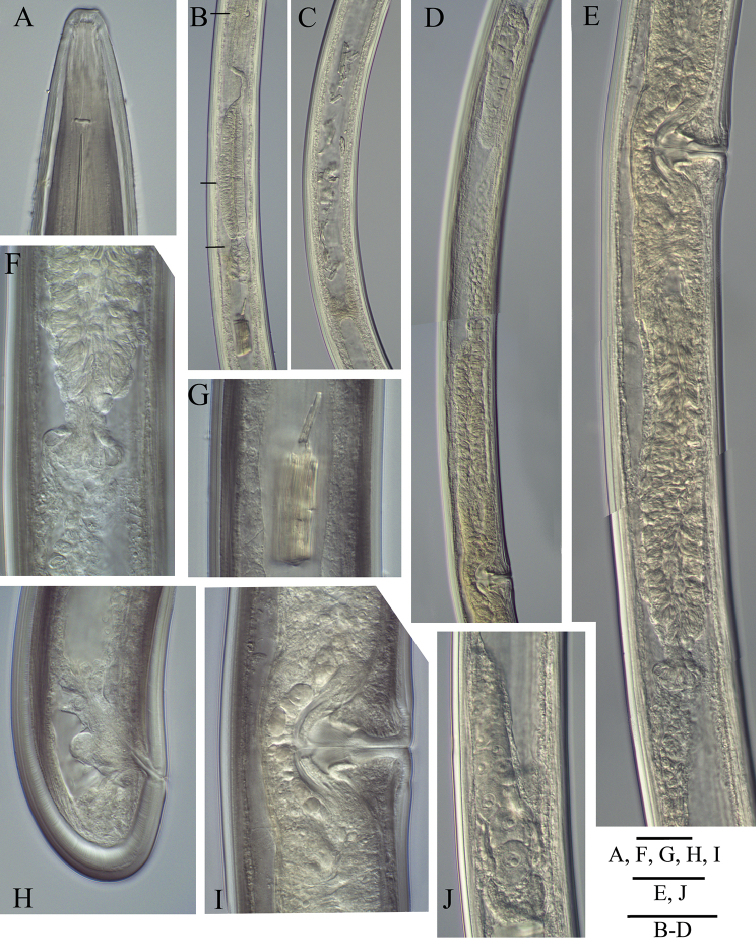
*Longidoruspolyae* sp. n., holotype **A** anterior end **B** pharyngeal region, arrows point to at nerve ring, pharyngeal bulb and cardia **C** intestine, posterior part with inclusions **D** anterior genital branch **E** vagina and part of the posterior genital branch **F** sphincter **G** intestine inclusion at higher magnification **H** tail **I** vagina **J** posterior ovary. Scale bars: 20 μm (**A, F–I**); 40 μm (**E, J**); 100 μm (**B–D**).

**Figure 4.**
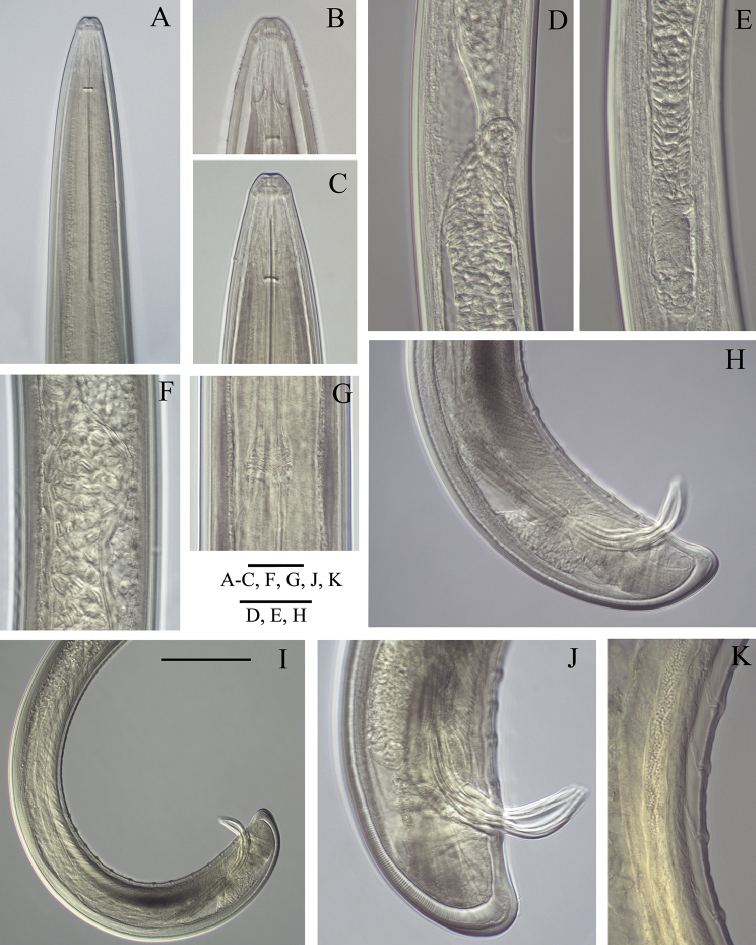
*Longidoruspolyae* sp. n., male **A** anterior region **B** amphidial fovea **C** labial region **D** junction of two testes **E** distal part of testis **F** sperm cells **G** nerve ring **H, I** posterior end, different magnifications **J** tail and spicules **K** supplements. Scale bars: 20 μm (**A–C, F, G, J, K**); 40 μm (**D, E, H**); 100 μm (**I**).

**Figure 5.**
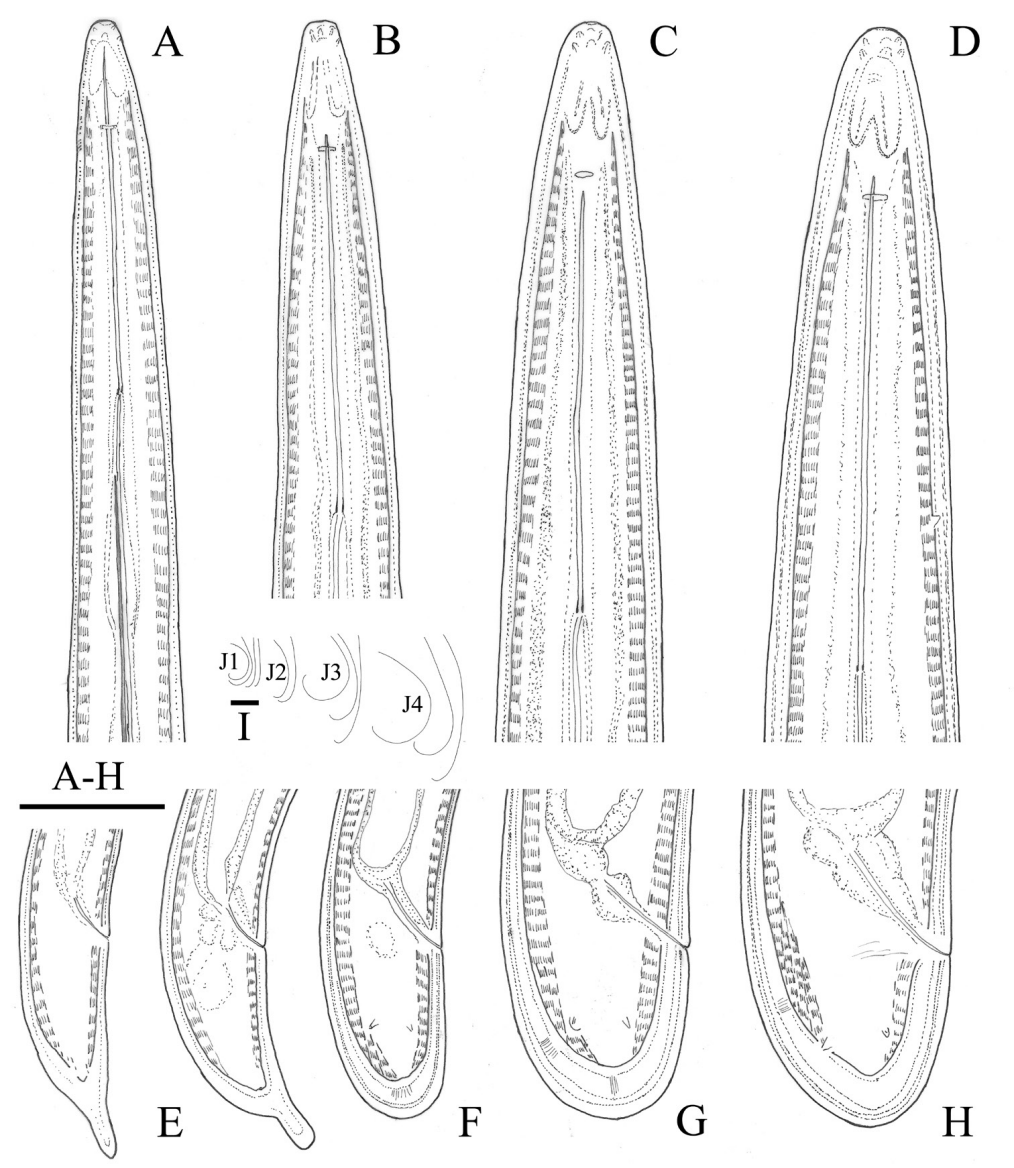
*Longidoruspolyae* sp. n. **A–D** anterior end of first- (J1) to fourth- (J4) stage juveniles **E–H** tail of first to fourth juvenile stages **I** variations in J1-J4 habitus shapes. Scale bars: 25 μm (**A–H**); 1 mm (**I**).

**Figure 6.**
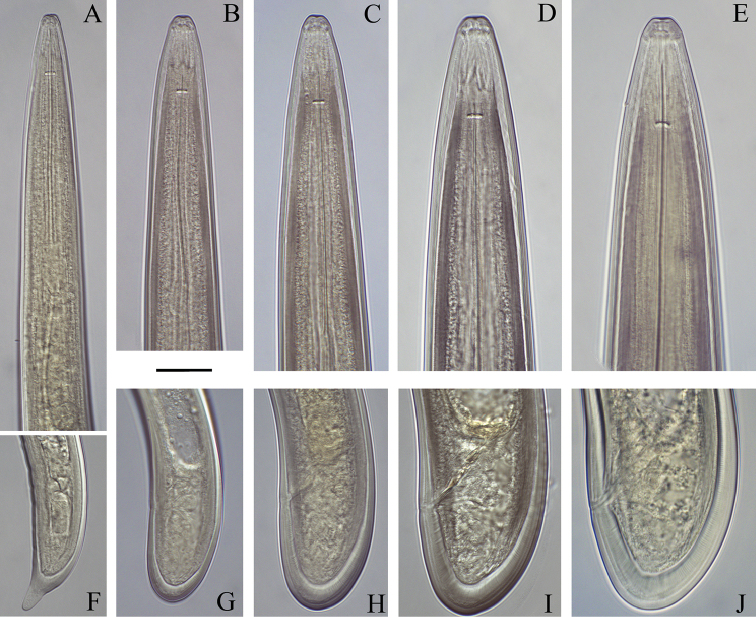
*Longidoruspolyae* sp. n. **A–E** anterior end of first- to fourth-stage juveniles and female **F–J** tail of first- to fourth- stage juvenile and female. Scale bar: 20 μm.

**Figure 7.**
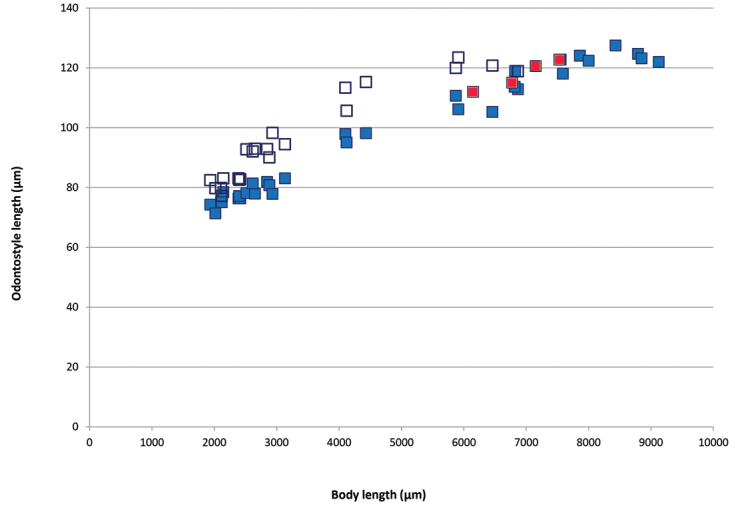
Scatter plot of functional odontostyle (blue square) and replacement odontostyle (white square) against body length of *Lonigidoruspolyae* sp. n. juveniles (J1-J4) and females (blue), and male odontostyle (red).

(for measurements see Table 1, Figs 1–7)

*Female.* Body assuming a spiral shape. Lip region narrow, 5–6 μm high, continuous with body profile, anteriorly flattened. Labial papillae, especially second circle, prominent, changing slightly the body contour. Cuticle 4–5 μm thick at postlabial region, 4–5 μm along the body, and 9–10 μm on tail posterior to anus. Guide ring 4–6 μm wide. Body pores conspicuous, 1 lateral pore anterior to or at the level of guide ring, 2 or 3 along odontostyle, 1 or 2 along odontophore, 3–5 in narrow part of the pharynx and 2–4 in bulb region as well as none dorsal and 5–6 ventral in pharyngeal region; numerous lateral pores observed along the rest of body. Amphidial fovea prominent, deeply bilobed, lobes long, slightly asymmetrical, amphidial aperture assumed to be a minute pore, hardly visible under light microscope. Odontostyle very slender, 1.5–2 μm wide at base. Two nerve rings observed, the first at some distance behind the odontophore and at 246.3 ± 11.1 (230–254) μm from anterior end, the second, more prominent, behind the first one at 354.8 ± 48.1 (327–440) μm from anterior end. Pharyngo-intestinal valve broadly rounded. Normal arrangement of pharyngeal glands: nuclei of the dorsal and ventrosublateral glands situated at 22.7–28.5 % and 54.3–58.5 % (*n* = 5) of the distance from anterior end of the bulb. Dorsal gland nuclei 2–2.5 μm in diameter, subventral gland nuclei 3–4 μm in diameter. In odontophore area of one female a small rudimentary odontostyle tip (vestigium) observed pointing forward. Peculiar crystalloid bodies of various sizes and shapes (mostly rod-like) found in the intestine of all females. Prerectum 465–497.5 μm long and rectum 33–42 μm or 0.6–0.8 of body diameter at anus. Tail bluntly conoid, rounded to hemispherical. Two pairs of lateral caudal pores. Vagina extending to *ca.* half the corresponding body width. *Pars distalis vaginae* 20–26 μm long; *pars proximalis vaginae* 21–27 μm long, thick walled. Uterus bipartite, moderately long, anterior uterus 230–406, posterior uterus 235–394 μm long, respectively; well-developed sphincter between uterus and *pars dilatata oviductus*, *pars dilatata*, and uteri containing numerous sperm cells; ovary small.

*Male.* Rarer than females. Habitus as in females, posterior part more strongly coiled ventrally. Shape of lip region similar to that in females. Cuticle 4.0–4.5 μm thick at postlabial region, 4 μm along the body and 5 μm on tail posterior to cloaca. One lateral pore anterior to or at the level of guide ring, 2 along odontostyle, 1 or 2 along odontophore, 3 or 4 in narrow part of the pharynx and 2 or 3 in pharyngeal bulb region, no dorsal and 6 ventral pores; numerous lateral pores present along the rest of the body. Two nerve rings observed, the first just behind the odontophore at a distance of 240.7 ± 12.0 (229–253) μm from anterior end, the second, more prominent behind the first one at 324 ± 12.7 (307.5–333) μm from anterior end. A small rudimentary odontostyle tip (vestigium) pointing forward observed in all males; in odontophore area (in 2 specimens) and in the slender part of pharynx (in 2 specimens). Pharyngo-intestinal valve broadly rounded. Tail short, bluntly conoid, dorsally convex, ventrally first straight then slightly concave. Three pairs of lateral pores on tail. One adclocal pair preceded by a row of 10 or 11 ventromedian supplements. Spicules slender, curved ventrally, lateral guiding piece sigmoid, 20–22 μm long. Spermatozoids oval (6–8 μm long).

*Juveniles*. Four juvenile stages can be differentiated based on the body, odontostyle, and replacement odontostyle length (Figs 5–7, Table 1). Habitus more or less an open C-shape, tail of the first stage juvenile digitate, with 9–11 μm long ventral peg, whereas in the subsequent developmental stages, bluntly rounded, **c**’ decreasing.

**Table 1.** Measurements of adults and juveniles of *Longidoruspolyae* sp. n. (mean ± standard deviation, with range). All measurements in micrometers except body length (mm).

**Character**	**Holotype**	**Females**	**Males**	**J4**	**J3**	**J2**	**J1**
		n = 10	n = 4	n = 8	n = 3	n = 7	n = 8
L	7.87	7.98 ± 0.82 6.81–9.12	6.90 ± 0.590 6.15–7.53	6.28 ± 0.476 5.87–6.87	4.10, 4.12, 4.43	2.80 ± 0.21 2.51–3.13	2.19 ± 0.19 1.9–2.4
a	119.5	105.5 ± 8.4 95.7–119.5	115.3 ± 9.8 101.9–125.1	102.3 ± 11.2 89.4–116.6	93.8, 96.9, 99.3	83.1 ± 3.4 77.1–87.2	77.1 ± 5.8 70.4–88.2
b	14.0	14.7 ± 1.5 2.3–17.3	12.2 ± 0.8 11.1–13.1	11.5 ± 0.4 11.2–12.0	8.8, 8.8, 9.0	7.6 ± 0.5 6.9–8.6	6.6 ± 0.9 5.5–8.0
c	206.9	211.3 ± 21.5 184.8–260.0	171.4 ± 25.4 146.3–197.4	155.0 ± 9.2 145.0–166.4	100.2, 96.7, 102.1	77.2 ± 4.3 70.8–83.3	48.0 ± 8.1 38.0–65.0
c’	0.7	0.7 ± 0.04 0.7–0.8	0.8 ± 0.1 0.8–0.9	0.8 ± 0.0 0.8–0.9	1.1, 1.1, 1.1	1.3 ± 0.0 1.3–1.4	2.3 ± 0.3 1.8–2.6
V (%)/ Spicules	51	50.9 ± 0.9 49.2–52.6	72.8 ± 1.9 71–74.5				
G1 (%)	8.1	8.0 ± 1.0 6.7–10.3					
G2 (%)	8.2	7.8 ± 1.0 6.0–9.6					
d	2.71	2.8 ± 0.1 2.7–2.9	2.4, 2.8				
d’	2.04	2.1 ± 0.1 2.0–2.2	1.8, 2.0				
Odontostyle	124	121.8 ± 3.9 114–127.5	117.6 ± 5.0 112–123	108.8 ± 3.6 105–113	98, 95, 98	80.2 ± 2.1 78–83	75.6 ± 2.1 71–78.5
Replacement odontostyle				120.8 ± 2.0 119–123.5	113, 106, 115	93.4 ± 2.5 90–98	81.4 ± 2.2 77–83
Odontophore	79	79.8 ± 5.2 68–84	80.2 ± 3.8 76–84	71.2 ± 8.2 65–77	54.5, 64, 68	56.7 ± 3.9 51–61	
Anterior end to guide ring	40	40.4 ± 1.4 37–42	39.4 ± 2.5 37–42	34.5 ± 2.6 32–38	32, 29.5, 31	25.4 ± 1.5 23–27	21.2 ± 0.9 20–23
Pharyngeal bulb length	155	152 ± 10.2 132–167	145.5 ± 3.0 142–148	139.6 ± 1.1 138–141	128, 115, 113	99.5 ± 2.8 95–102	84.1 ± 6.0 78–94
Pharyngeal bulb width	29	26 ± 1.1 25–28	24.8 ± 1.7 23–27	23.6 ± 1.3 22–25	23, 19, 20	18.25 ± 0.5 17–19	14.7 ± 1.2 14–17
Developing gonad	–	–	–	73.7 ± 9.9 7–85	35, 33, 35	26.6 ± 1.6 22–30	19.6 ± 2.4 17–23
Pharynx	562	553.5 ± 54.1 481–646	565.0 ± 36.5 518–601	554.6 ± 26.1 524.5–571	468, 466, 494	367.9 ± 16.7 342–387	328.9 ± 20.9 298–359.5
Tail	38	37.9 ± 3.0 33–43	41.0 ± 7.3 1–47	40.6 ± 3.5 37.5–45.5	41, 43, 43	36.2 ± 1.5 35–38	46.4 ± 6.2 2–52
Length of hyaline part of tail	12	12.3 ± 0.7 12–13	12.5 ± 2.5 11–14				
**Body diameter at**:							
– lip region	14.5	14.6 ± 0.5 4–15.5	14.8 ± 0.6 14–15	12.2 ± 1.0 11–13	11, 11	8.2 ± 0.6 7–9	6.8 ± 0.2 6.5–7
– guide ring	30	30.3 ± 1.0 29–32	29.4 ± 2.0 27–32				
– base of pharynx	61	63.1 ± 4.7 59–75	56.0 ± 2.7 52–58	54.2 ± 1.2 52.5–55	43, 42, 44	33.5 ± 1.6 31–35.5	27.1 ± 2.0 24.5–30
– mid-body/vulva	66	76.0 ± 8.6 66–92.5	60.0 ± 5.5 53–66.5	61.8 ± 6.1 55–68	44, 42.5, 45	33.6 ± 1.5 32–36	28.6 ± 3.6 24–34
– anus	53	53.1 ± 3.6 49– 61	48.2 ± 5.5 41–53	51.3 ± 1.7 49–53	36.5, 39, 40	27.1 ± 1.5 25–29	20.6 ± 2.2 18–24
– hyaline part	35	37.4 ± 3.1 35–42	26.5 ± 1.2 26–27				

#### Type locality and plant association.

Balgarene village, Pre-Balkan zone of the Balkan Mountains, north-central Bulgaria (43°2'48.08"N; 24°46'24.53"E), 386 m a.s.l., private orchard, rhizosphere of *Pyruscommunis* L.

#### Type material.

The holotype (PNT 42), 20 paratype females, 2 males, and 92 juveniles (overall PNT 43-101) from all stages are deposited in the nematode collection of the Institute of Biodiversity and Ecosystem Research, BAS, Sofia, Bulgaria; 1 female, 1 male and 6 juveniles in the USDA Nematode Collection, Beltsville, Maryland, USA; 1 female, 1 male and 6 juveniles in the Wageningen Nematode Collection (WANECO), Wageningen, the Netherlands; 1 female and 8 juveniles in the Nematode Collection of the Institute of Sustainable Plant Protection, Bari, Italy.

#### Molecular characterisation.

The NCBI BLASTn search of D2-D3 expansion segments of the 28S rRNA gene sequence showed highest similarity (93–94%) to several *Longidorus* species (*L.attenuatus* Hooper, 1961; *L.dunensis* Brinkmam, Loof & Barbez, 1987; *L.athesinus* Lamberti, Coiro & Agostinelli, 1991; *L.persicus* Esmaeili, Heydari, Archidona-Yuste, Castillo & Palomares-Rius, 2017; *L.euonymus* Mali & Hooper, 1974; *Longidorus* sp. 1, and *Longidorus* sp. 3). The highest identity (93.0–93.1%) was calculated with populations identified as *L.attenuatus* and *L.dunensis* (accessions KT755457 and AY593057, respectively). However, in the 28S rDNA phylogenetic analyses *L.polyae* sp. n. grouped in a clade with four *Longidorus* spp. (*L.athesinus*, Italy; *Longidorus* sp. 1, *Longidorus* sp. 2, and *Longidorus* sp. 6 from USA) with intermediate to high PP support (0.7–1.0) depending of the applied MSA algorithm (Fig. 9). The phylogenetic position of the new species based of 18S rRNA gene remained unresolved (Fig. 10) and was changing when different MSA algorithms and outgroups were tested. The pairwise sequence comparisons revealed highest identity (99.2%) with 18S rDNA sequences of *L.attenuatus* (AY687994), *L.elongatus* (de Man, 1876) Micoletzky, 1922 (EU503141), *L.piceicola* Liskova, Robbins & Brown, 1997 (AY687993), and *L.uroshis* Krnjaić, Lamberti, Krnjaić, Agostinelli & Radicci, 2000 (EF538760) (or 1564 identical residues of 1577 MSA length).

#### Diagnosis and relationships.

*Longodoruspolyae* sp. n. is a comparatively large bisexual species average 7.98 (6.81–9.12 mm) with the odontostyle over 100 μm (114.0–127.5 μm) long; the lip region narrow (14.0–15.5 μm), almost continuous with body profile, anteriorly flat; amphidial fovea long, pocket-shaped, deeply bilobed, and lobes slightly asymmetrical; normal arrangement of pharyngeal glands; and tail short, bluntly rounded to hemispherical, four juvenile stages present, with the tail of the first-stage juvenile digitate.

The alpha-numeric codes for *L.polyae* sp. n. to be applied to the polytomous identification key for *Longidorus* species by Chen et al. (1997) and partial polytomous key proposed by Peneva et al. (2013) are: A5, B2, C34, D3, E3, F45, G12, H1, I2, J1, K7 (Table 2).

**Table 2.** A partial polytomous key to the species of *Longidorus* species close to *L.polyae* sp. n., based on the key by Chen et al. (1997) and Peneva et al. (2013) incorporating species described after 1997.

*Longidorus* species	A	B	C	D	E	F	G	H	I	J	K
***L.polyae* sp. n.**	**5**	**2**	**34**	**3**	**3**	**45**	**12**	**1**	**2**	**1**	**7**
* L. arthensis *	4	23	3	1	2	3	12	12	2	1	67
* L. pauli *	4	23	23	3	23	4	3	1	2	1	7
* L. silvae *	45	23	34	13*	3	34	2(3)	11	1	1	7
* L. kheirii *	45	345	34	13*	2	34	12	1	2	1	7
* L. uroshis *	56	24	34	3	23	34	2	1	2	1	7

Note: A – odontostyle length; B – lip region width; C – distance of guide ring to anterior body length; D – shape of anterior region; E – amphidial fovea shape; F – body length; G – index “**a**”; H – tail shape; I – presence/absence of male; J – number of juvenile stages; K – tail shape in first stage juvenile. *Changes in code D proposed.

The group of large *Longidorus* species (code F34) with a moderately long odontostyle (code A45), pocket-shaped amphidial fovea, bilobed, symmetrical (code E2) or asymmetrical (code E3), normal arrangement of pharyngeal glands nuclei, short rounded tail (code H1) and digitate tail or tail with mucro (code K7) (according to Peneva et al. 2013) consists of a few species, namely: *L.arthensis*; *L.pauli* Lamberti, Molinari, De Luca, Agostinelli & Di Vito, 1999; *L.kheirii* Pedram, Niknam, Robbins, Ye & Karegar, 2008; *L.silvae* Roca, 1993; and *L.uroshis* (Table 2). The new species differs from these by the presence of peculiar inclusions in the intestine. Furthermore, it differs from:

*L.pauli* by females having differently shaped amphidial pouches (asymmetrically vs symmetrically bilobed); plumper body (**a** = 95.7–119.5 vs 120.3–143.5); more posterior guide ring position (37–42 vs 27–36 μm); longer spicules (71.0–74.5 vs 61–69 μm); guiding piece in male sigmoid vs straight; different shape of tail in second- and third-stage juveniles (bluntly rounded vs conical) (Lamberti et al. 1999);

*L.uroshis* by females having a longer body (6.81–9.12 vs 5.6–7.6 mm); narrower lip region (average 14.6 (14.0–15.5) vs average 17 (15.0–20.5) μm), higher **c** (184.8–260.0 vs 120.4–162.0) and lower **c**’ values (0.7–0.8 vs 0.9–1.0) (Krnjaić et al. 2000);

*L.kheirii* by females having a narrower lip region (14.0–15.5 vs 19.5–23.0 μm) and pharyngeal bulb (25–29 vs 39.5–48 μm), higher **c** values (184.8–260.0 vs 119.0–167.8), amphidial pouches deeply bilobed *vs* not to slightly bilobed; differently shaped tail of the first- and second-stage juvenile and different ovarium structure (Pedram et al. 2008).

*L.arthensis* by females having longer body (6.81–9.12 vs 5.14–6.74 mm) and odontostyle (114.0–127.5 vs 102.0–111.0 μm); asymmetrically vs evenly bilobed amphidial pouches, lower **c**’ values (0.7–0.8 vs 1.0); males with longer spicules (71.0–74.5 vs 60.0–66.0 μm) (Brown et al. 1994);

*L.silvae* by females having two vs one nerve rings, differently shaped tail of the first-stage juvenile (subcylindrical, rarely cylindrical part/mucro with ventral position vs cylindrical mucro with central position), mucro shorter (9–11 vs 20–27 μm), higher **c** values (average 211.3 (184.8–260.0) vs average 166.7 (132.0–189.0) and posteriorly located vulva (50.9 (49.2–52.6) vs 48.6 (44.9–50.7) (Roca 1993). Males common vs absent (Roca 1993) or rare (Barsi and Lamberti 2004; Barsi et al. 2007).

Additionally, it can be differentiated from *L.athesinus*, a phylogenetically related species (Fig. 9), by females having longer body (6.81–9.12 vs 3.7–5.8 mm) and odontostyle (114.0–127.5 vs 83.5–94.0 μm); higher **a** value (95.7–119.5 vs 56.2–88.1); differently shaped tail in first-stage juvenile (digitate vs bluntly conoid) (Lamberti et al. 1991);

Morphometrical data of the most similar species are presented in Table 3.

**Table 3.** Morphometric comparisons of *Longidoruspolyae* sp. n. and related *Longidorus* spp. with similar morphological characters and DNA sequences.

Species	L (mm)	a	c’	Odontostyle length (µm)	Lip region width (µm)	Guide ring position (µm)
*** L. polyae ***	**6.81–9.12**	**95.7–119.5**	**0.7–0.8**	**114–127.5**	**14–15.5**	**37–42**
* L. arthensis *	5.14–6.74	74.5–110	0.8–1.1	102–111	14–17	30–38
* L. athesinus *	3.7–5.8	56.2–88.1	0.7–1.1	83.5–94	14–18	32–38
* L. kheirii *	6.7–9.0	60.3–82	0.6–0.9	113–130	19.5–23	36.5–45
* L. pauli *	6.5–8.6	120.3–143.5	0.8–1.0	102–118	14–17	27–36
* L. silvae *	5.9–8.0	87.5–123.5	0.72–0.84	113.5–133.0	14.0–17.0	37.0–44.0
* L. uroshis *	5.6–7.6	96.9–108.9	0.9–1.0	125–144	15–20.5	38–47

Note: Decimals are omitted for measurements in μm.

#### Etymology.

Named after the first author’s sister Mrs Polya Kadiyska, a school teacher of art and iconography at the “Nikola Obretenov” Primary school in Rousse, Bulgaria.

### Longidorus pisi

Taxon classificationAnimaliaDorylaimidaLongidoridae

Edward, Misra & Singh, 1964

= Longidoruslatocephalus Lamberti, Choleva & Agostinelli, 1983

#### Notes.

**Figure 8.**
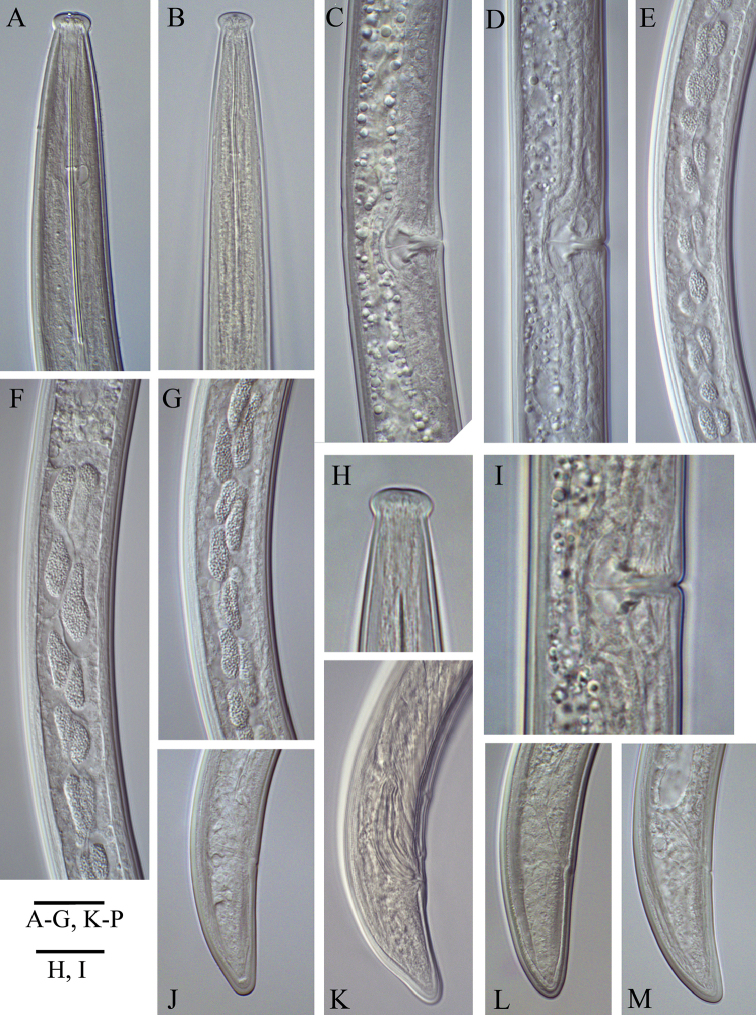
Bulgarian populations of *Longidoruspisi* Edward, Misra & Singh, 1964, anterior end of female (**A)** and male (**B**) female **C, D** vagina and part of reproductive system (different populations) **E–G** pre-rectum inclusions **H** labial region **I** vagina **J–M** variations in tail shape, female (**J, L, M**) and male (**K**). Populations: **A, L** Petrich **C, E–G, M** Sandanski **D, H, I, J** Kromidovo **B, K** male specimens from a tobacco field, Petrich region. Scale bars: 20 μm (**A–G, J–M**); 12 μm (**H, I**).

Morphological and morphometric data for females and juvenile stages are presented in Table 4 and in Figure 8. The morphometric data obtained in this study agreed with those of *L.latocephalus* from its type locality (Lamberti et al. 1983) and several additional populations studied by Lamberti et al. (1997). Furthermore, when compared to the type population of *L.pisi* (Edward et al. 1964), specimens from our populations revealed longer odontostyle (average 75 (72–79) and average 76 (74–78) vs 58 (56–61) μm) and odontophore (average 50 (45–53) and average 49 (46–53) vs average 42.7 (35–43) μm); longer distances anterior end to guide (average 44 (42–46) vs average 32 (31–35) μm) and nerve ring (average 147 (138–151) and 141 (132–150) vs average 133 μm); wider (average 10.6 vs 7.5 μm) and higher (5–6 vs 3.5 μm) lip region and lower **c**’ ratio (average 1.9 (1.5–2.0) and average 1.7 (1.5–1.8) vs average 2.5 (2.4–2.6). The morphometrics of our populations were more similar to the Iranian population of *L.pisi* (Saveh, Markazi province) for which a D2-D3 expansion domain of 28S rRNA gene sequence identical to ours is available (Pedram et al. 2012). Differences in a few characters were observed, e.g. smaller **a** (average 125.2 (117.2–132.1) and 127.3 (119.8–132.9) vs 139.4 (134.8–144.6) and **c**’ (average 1.9 (1.5–2.0) and average 1.7 (1.5–1.8) vs average 2.5 (2.3–2.9) values, larger diameter at anus level (average 22.9 (21–24.5) and average 20.5 (20–22) vs average 18.1 (16–19) μm) and slightly shorter tail (average 38.9 (36–43) and average 39.1 (37–42) vs average 45 (43–47) μm) compared to the latter population.

**Table 4.** Measurements of adults and juveniles of *Longidoruspisi* (mean ± standard deviation, with range) from different crops in Bulgaria. All measurements in micrometers except body length (mm).

Host Character	Petrich	Sandanski	Kromidovo	Petrich		
	* Nicotiana tabacum *	* Vitis vinifera *		* Nicotiana tabacum *		
	female	female	female	J1	J2	J3
	n = 9	n = 6	n = 2	n = 2	n = 9	n = 2
L	4.02 ± 0.2 (3.77–4.28)	3.73 ± 0.2 (3.37–3.91)	3.17, 3.91	1.10, 1.21	1.8 ± 0.2 (1.55–2.07)	2.71, 2.65
a	125.2 ± 6.3 (117.2–132.1)	127.3 ± 5.2 (119.8–132.9)	113.7, 129.5	68.7, 68.6	82.2 ± 4.7 (75.8–88.6)	104.0, 99.1
b	12.1 ± 0.9 (10.9–13.4)	11.8 ± 1.2 (10.3–13.2)	8.8, 11.9	5.0, 5.7	6.8 ± 0.7 (5.9–8.2)	9.5, 9.1
c	102.8 ± 4.2 (96.7–111.3)	95.5 ± 5.7 (87.2–102.0)	80.9, 94.7	34.1, 38.2	46.2 ± 3.2 (41.2–50.3)	54.6, 63.7
c’	1.7 ± 0.1 (1.5–1.8)	1.9 ± 0.11 (1.7–2.0)	2.0, 2.0	3.0, 2.6	2.7 ± 0.2 2.5–2.9)	2.4, 2.2
V (%)	48.4 ± 0.9 (47.0–49.7)	50.9 ± 0.6 (50.2–51.7)	51.4, 51.4			
G1 (%)	5.3 ± 0.6 (4.9–6.2)					
G2 (%)	5.7 ± 0.6 (5.2–6.6)					
Odontostyle	74.8 ± 2.3 (72–79)	76.1 ± 1.7 (74–78)	75, 78	47.5, 46.5	52.9 ± 1.3 (50–55)	59, 59
Replacement odontostyle				53, 53	63.2 ± 1.5 (61–65)	76, 77
Odontophore	49.8 ± 2.8 (45–53)	49.4 ± 2.8 (46–53)	50		39.3 ± 2.1 (37–42)	
Anterior end to guide ring	44.1 ± 1.6 (42–46)	43.6 ± 0.9 (43–45)	44, 42	25, –	31.7 ± 1.4 (30–34)	36, 36
Pharyngeal bulbus length	69.1 ± 2.9 (65–73)	71.3 ± 1.4 (69.5–74)	76, 74	45, 37	51.5 ± 3.3 (47–56)	63, 62
Anterior to nerve ring	147.4 ± 5.0 (138–151)	141.4 ± 6.2 (132–150)	–, 153		115.4 ± 4.4 (110–121)	130, 138
Pharynx	330.9 ± 20.8 (292–368)	310.7 ± 23.3 (291–342)	361, 328	222, 212	263.4 ± 14.8 (242–285)	286, 292
Tail	38.7 ± 2.0 (36–43)	39.1 ± 1.8 (37–42)	39, 41	32, 32	39 ± 2.5 (35–43)	50, 42
Body diameter at:						
– lip region	11.0 ± 0.5 (10–12)	10.3 ± 0.3 (10–11)	10, 11	7, 7	8.5 ± 0.9 (8–9)	10.5, 9
– base of pharynx	27.9 ± 0.8 (27–29)	25.6 ± 0.5 (25–26)	27, 26.5	16, 17	20.5 ± 0.7 (19.5–22)	26, 24.5
– mid-body	31.9 ± 1.1 (30–34)	29.3 ± 0.8 28–30)	28, 30	16, 18	21.7 ± 0.9 (20–23.5)	26, 27
– anus	22.9 ± 1.2 (21–24.5)	20.5 ± 0.8 (20–22)	20, 21	11, 12	14.5 ± 0.5 (14–15)	21, 19

#### Sequence and phylogenetic analyses.

Three ribosomal DNA regions (D2-D3 expansion segments of 28S rRNA gene, 18S rRNA gene, and ITS1-5.8S-ITS2 regions) of *L.pisi* were amplified and sequenced. The D2-D3 expansion segments of 28S rRNA gene sequences from all populations were identical to that of the Iranian population (JQ240274, Pedram et al. 2012) and differed slightly (1 and 3 bp) from those of other populations (Greece (AY601569) and South Africa (AY601568), respectively) identified as *L.latocephalus* (He et al. 2005). In the phylogenetic analysis, all aforementioned sequences formed a clade with maximal Bayesian posterior probability (1.0) and showed a close relationship with *L.mindanaoensis*. The BLASTn search using 18S rRNA gene sequence revealed highest identity (99%) with five accessions (two *Longidorus* (HQ735099*L.mindanaoensis* Coomans, Tandingan De Ley, Angsinco Jimenez & De Ley, 2012 and AY283163*L.ferrisi* Robbins, Ye & Pedram, 2009) and three *Paralongidorus* species (JN032586*P.bikanerensis* (Lal & Mathur, 1987) Siddiqi, Baujard & Mounport, 1993; AJ875152*P.maximus* (Bütschli, 1874) Siddiqi, 1964 and KJ427794*P.rex* Andrássy, 1986). A pairwise comparison of *L.pisi* sequence with the closest sequences (AY283163, HQ735099, JN032586, AJ875152 and KJ427794) revealed 14–22 different nucleotides. The 18S rDNA phylogenetic tree is presented in Figure 10.

**Figure 9.**
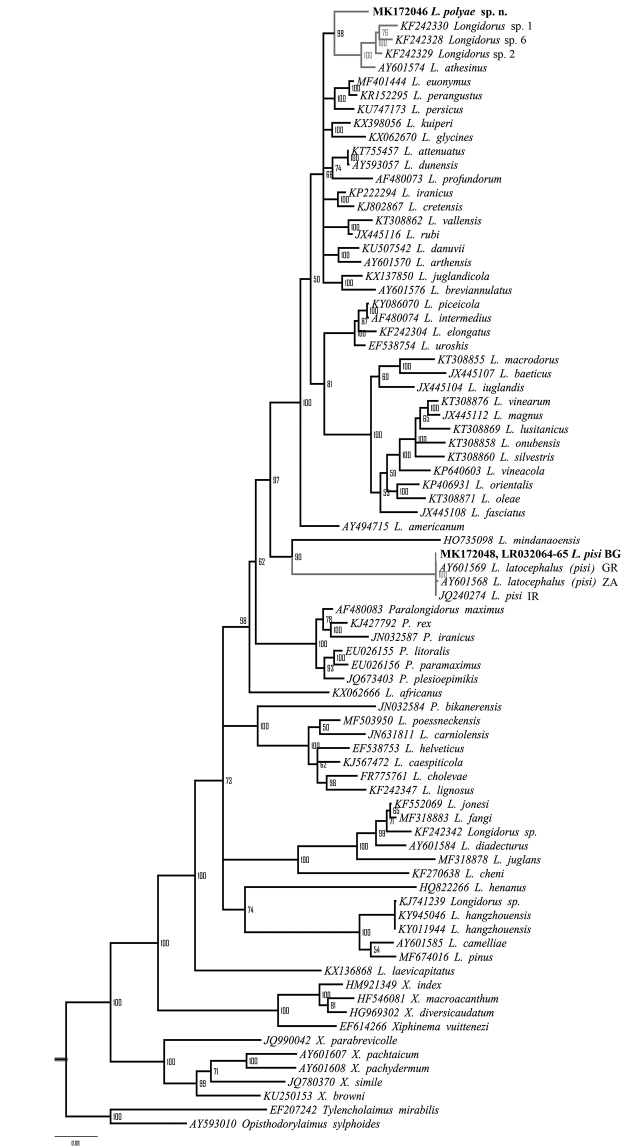
Phylogenetic tree using D2-D3 expansion segments of the 28S rRNA gene inferred from a Bayesian analysis with GTR+I+G model. Numbers represent the Bayesian posterior probabilities.

**Figure 10.**
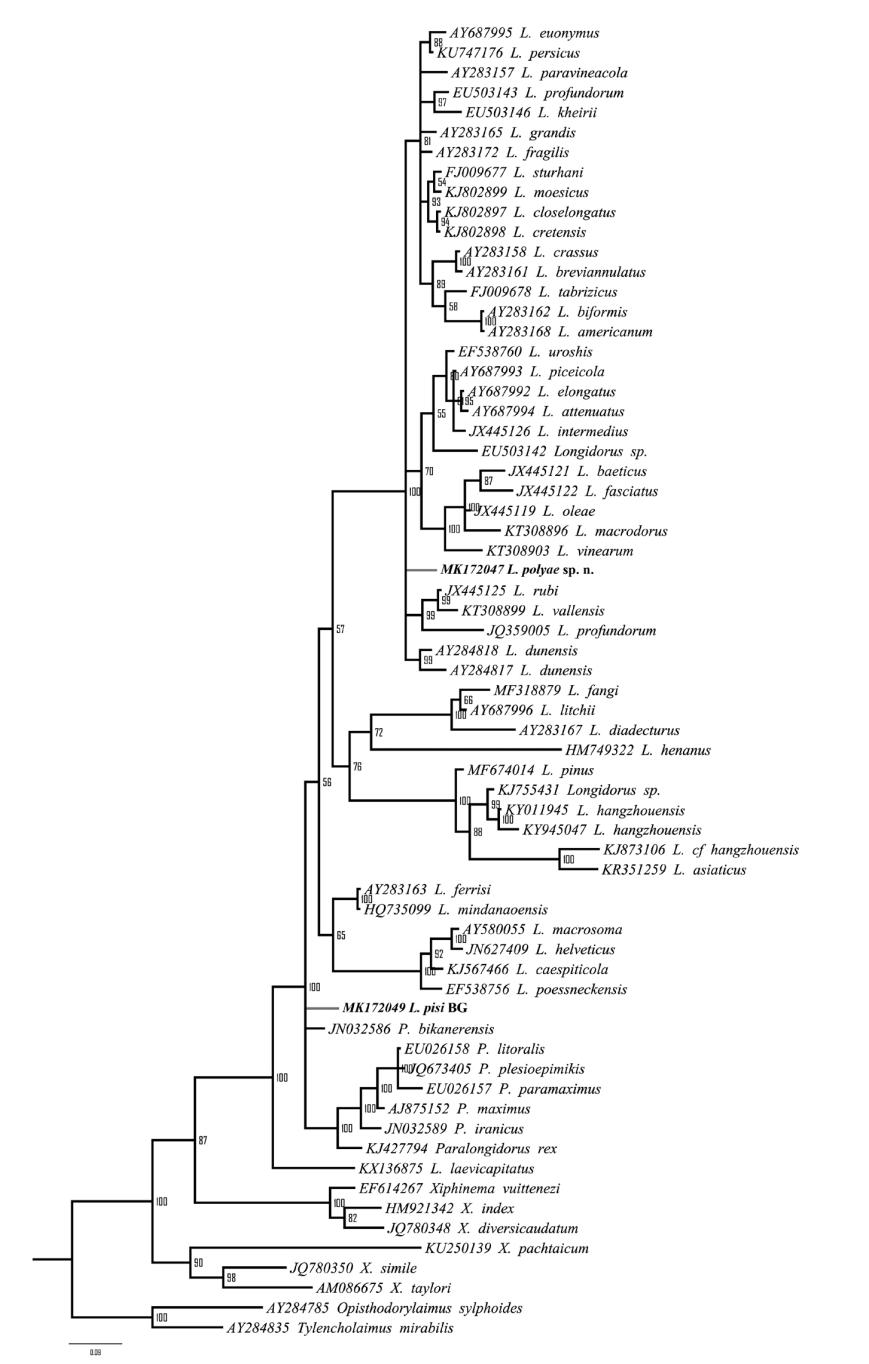
Phylogenetic tree using 18S rRNA gene inferred from a Bayesian analysis with GTR+I+G model. Numbers represent the Bayesian posterior probabilities.

## Discussion

### *Longidoruspolyae* Lazarova, Elshishka, Radoslavov & Peneva sp. n.

The new species belongs to a group of large *Longidorus* species having a moderately long odontostyle, a bilobed pocket-shaped amphidial fovea, a short rounded tail in adults, and a digitate tail with mucro in first-stage juveniles. The group contains just a few species occurring in Europe (*L.arthensis*, *L.silvae*, *L.uroshis*), Syria (*L.pauli*), and Iran (*L.kheirii*). A very specific character observed in all female specimens of *L.polyae* sp. n. were the crystalloid structures of various shapes and sizes present in the intestinal lumen, especially in the posterior gut region. To our knowledge, the presence of similar crystalloid structures has been rarely observed in plant-parasitic species (e.g. *L.perangustus* (Roshan-Bakhsh et al. 2016) and *Hirschmanniellakwazuna* (Van den Berg et al. 2009)), and more often in non-parasitic nematodes (Heyns and Coomans 1983; Bird et al. 1991; Tahseen 2012; Abebe et al. 2013).

DNA data were available for all species except *L.pauli*. However, different ribosomal fragments have been deposited in the GenBank database, which limit the reconstruction of phylogenetic relationships within the group. Moreover, the D2-D3 expansion segments of 28S rDNA sequences of the new species showed highest similarity (93%) to two other species of *Longidorus*, *L.attenuatus* and *L.dunensis*, with sequences revealing a very high inter-species similarity (or 1–3 nucleotides difference). One population of *L.silvae* from Serbia, characterised molecularly by three sequences of D3 segment of 28S rDNA (AM412367-AM412369, Barsi et al. 2007), was very similar to *L.polyae* sp. n. comparing the same fragment (2 nucleotides difference at the 3’ end, overall multiple sequence alignment of 300 characters). Likewise, a very high level of similarity for the same ribosomal segment of 28S rDNA are known for other *Longidorus* species, e.g. the multiple alignment of 10 *L.aetnaeus* Roca, Lamberti, Agostinelli & Vinciguerra, 1986 and five *L.leptocephalus* Hooper, 1961 sequences revealed one indel for an alignment length of 237 characters. However, both species showed higher inter-species dissimilarity in D2-D3 expansion segments of 28S rDNA (0.7–1.6%) and the *cox*1 mtDNA region (3.2%) (Palomares-Rius et al. 2017).

In comparing the morphology of *L.polaye* sp. n. to the most closely related species, inaccuracies in code D values (related to the shape of anterior regions) were identified. In the original description of *L.silvae*, the lip region was described as “subacute, flattened frontally and continuous with the rest of the body” (Roca 1993: 211), which rather corresponds to D3 code description (“body tapering distinctly, lip region flattened, continuous, or slightly offset by depression”) than to D1 (“body tapering distinctly, lip region rounded, continuous”) according to Chen et al. (1997: 18). Similarly, in the diagnosis of *L.kheirii*, a D1 code was assigned instead of D3 (“head continuous with the contour of the rest of body, a truncate and slightly concave” Pedram et al. 2008: 206).

### *Logidoruspisi* Edward, Misra, & Singh, 1964

Morphological and molecular data of three Bulgarian populations of *L.pisi* have been presented within the framework of this study. Some differences in morphometrics between these populations and the original description have been observed; however, a more comprehensive integrative study on materials from the type area would help to evaluate the divergences. New sequences for two ribosomal DNA regions (18S rRNA gene and ITS1-5.8S-ITS2) were obtained for *L.pisi* for the first time. The D2-D3 expansion segments of 28S rRNA gene sequences of all populations from Bulgaria were identical. In the phylogenetic analysis (Fig. 9), our populations and all populations of *L.pisi* (= *L.latocephalus*) from Greece, Iran, and South Africa clustered together with *L.mindanaoensis* and thus supports their evolutionary relationship as revealed by a previous study (Coomans et al. 2012). However, in the phylogenetic tree based on the 18S rRNA gene (Fig. 10), the position of *L.pisi* was not resolved. Both phylogenetic trees showed incongruent evolutionary relationships between *L.pisi* and *L.mindanaoensis*. The ITS sequence of *L.pisi* showed no similarity with the corresponding region of *Longidorus* species present in the database and no phylogenetic analyses were carried out.

The species appears to have a wide distribution, having been reported from three continents and numerous countries: Asia (India, Pakistan, Iran, China), Africa (Botswana, Egypt, Malawi, Mozambique, Cameroon, Libya, Namibia, Senegal, South Africa, Sudan), and Europe (Bulgaria, Macedonia, Greece). It was associated mainly with cultivated plants (pea, grapevine, mint and other medical plants, maize, potato, apple, pear, sugarcane) and rarely with natural vegetation (Marais and Swart 2002; Bohra and Baqri 2005). In Bulgaria and other Balkan countries, it was found in soils from agricultural habitats, associated with annual and perennial crops (tobacco, tomato, sweet pepper, black currant, grapevine, apple, peach, kiwifruit walnut, sweet chestnut, *Pinusnigra* J.F. Arnold, etc.) (Lamberti et al. 1983; Choleva et al. 1991, 1997), which suggest its probable introduction to Europe.

## Supplementary Material

XML Treatment for Longidorus polyae

XML Treatment for Longidorus pisi
